# Optical Detection of SARS-CoV-2 Utilizing Antigen-Antibody Binding Interactions

**DOI:** 10.3390/s21196596

**Published:** 2021-10-02

**Authors:** Mahmoud Al Ahmad, Farah Mustafa, Neena Panicker, Tahir A. Rizvi

**Affiliations:** 1 Electrical Engineering Department, United Arab Emirates University, Al Ain 15551, United Arab Emirates; 2 Zayed Center for Health Sciences, United Arab Emirates University, Al Ain 15551, United Arab Emirates; fmustafa@uaeu.ac.ae (F.M.); tarizvi@uaeu.ac.ae (T.A.R.); 3 Department of Biochemistry, College of Medicine & Health Sciences, Al Ain 20000, United Arab Emirates; ngpanicker@uaeu.ac.ae; 4 Department of Microbiology and Immunology, College of Medicine & Health Sciences, Al Ain 20000, United Arab Emirates

**Keywords:** COVID-19, NC protein, optical detection, protein–protein interactions, RBD, SARS-CoV-2

## Abstract

Severe acute respiratory syndrome coronavirus 2 (SARS-CoV-2), the virus responsible for the coronavirus disease (COVID-19) pandemic, is sweeping the world today. This study investigates the optical detection of SARS-CoV-2, utilizing the antigen-antibody binding interactions utilizing a light source from a smart phone and a portable spectrophotometer. The proof-of-concept is shown by detecting soluble preparations of spike protein subunits from SARS-CoV-2, followed by detection of the actual binding potential of the SARS-CoV-2 proteins with their corresponding antigens. The measured binding interactions for RBD and NCP proteins with their corresponding antibodies under different conditions have been measured and analyzed. Based on these observations, a “hump or spike” in light intensity is observed when a specific molecular interaction takes place between two proteins. The optical responses could further be analyzed using the principle component analysis technique to enhance and allows precise detection of the specific target in a multi-protein mixture.

## 1. Introduction

The world is currently facing the COVID-19 pandemic, caused by the appearance of a novel coronavirus in the human population at the end of 2019 [1]. Within a few months, this virus had spread to most countries across the world, infecting millions (>21 million as of 17 August 2020) and causing >770,000 deaths [2]. Rapid detection methods, independent of lab settings, have been identified as top priorities in promoting epidemic prevention and control. Currently, the molecular technique of quantitative real time polymerase chain reaction (qRT PCR) is the gold standard for SARS-CoV-2 detection using samples from respiratory secretions [3,4,5,6,7]. However, this time-consuming and cumbersome procedure involves long processing times (days) for results [8]. Several other molecular assays have been developed to detect SARS-CoV-2, such as enzyme-based assays, such as ELISAs, and rapid tests that aim to detect either antibodies against the virus or the viral antigen themselves [4]. Nevertheless, most of these antigen-antibody-based assays have failed quality control due to their rapid development without proper testing, resulting in either false negative or false positive detection, due to the long time it takes to develop serum responses to the viral infection (from days to weeks) [9]. Thus, most of the methods used so far require skilled manpower, are time consuming if accurate, or not reliable at all, if fast. On the other hand, biosensor technology provides excellent sensitivity, but it has its own caveats. For example, some biosensors require metal coating deposited on the device, thereby raising costs [10], while others suffer from temperature-dependence, which can be a hindrance for portable biosensors in outdoor conditions [11]. Some require expensive reagents and reaction times that are often longer [12]. Mavrikou et al. have used bioelectric recognition assays along with artificially engineered cells to demonstrate direct detection of SARS-CoV-2 surface antigens without prior sample processing [13]; however, they still have to show whether their systems will work in a real-world scenario. Optical, label-free biosensors have been utilized frequently in biomolecular detection due to their continuous monitoring abilities, and high sensitivity to local variations, including the refractive index change [14]. They are capable of detecting interactions between molecules and their surrounding media [15].

**Figure 1:**
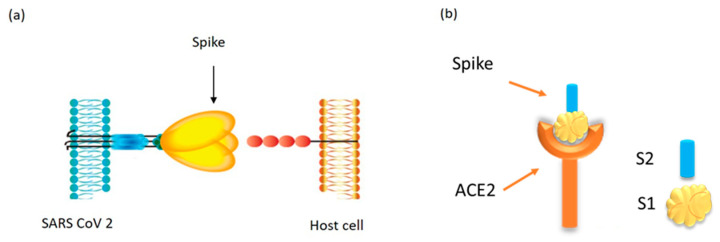
Schematic illustration of the SARS-CoV-2 spike protein and ACE2 receptor binding. (**a**) SARS-CoV-2 binding to the ACE2 receptor on the host cell surface. (**b**) Binding of ACE2 and the spike protein along with an illustration of the spike protein subunits, S1 and S2.

In terms of detection—the most prominent feature of the SARS-CoV-2 virus, like other coronaviruses, is the spike protein (S) that protrudes out of the virus particle, essentially like “spikes” as the name suggests. The spike protein forms a trimer that is used by the virus to enter susceptible cells using the angiotensin-converting enzyme 2 (ACE2) protein as the cellular receptor [16], the same protein used by the SARS-CoV-1 virus that caused the first SARS epidemic in 2003 (Figure 1a). The spike protein is cleaved by host proteases into two subunits: the surface subunit S1 and the transmembrane subunit S2 [17] (Figure 1b). The surface S1 subunit is used by the virus to interact with the ACE2 protein, using its receptor-binding domain (RBD) [18]. This allows the virus to attach to the susceptible cells, while the S2 protein is used for the actual fusion of the virus with the cell membrane, allowing the virus to be endocytosed and release its genomic RNA cargo, wrapped up in the nucleocapsid protein (NCP), into the cytoplasm [19,20]. The viral genomic RNA is immediately used to translate viral proteins that are used for successful virus replication in the susceptible cells [21]. The spike protein is also one of the most immunogenic proteins of the virus, towards which most of the neutralizing antibody responses against the virus are generated in infected individuals, making it an ideal candidate for a vaccine, as well as a target of drug development [22,23,24].

The receptor-binding domain of SARS-CoV-2 is the key region of the S protein that affects the virus spread. Xia et al. have confirmed these observations by showing that even the fusion capability of the SARS-CoV-2 S2 subunit is better than that of SARS-CoV-1, further explaining the increased infectivity of the virus compared with other coronaviruses [25,26]. They further show that lipopeptide inhibitors can be developed, which can disrupt such fusion capability to inhibit the ability of the virus to infect cells [27]. Similarly, Seydoux et al. have shown the utility of isolating S-specific antibody-producing B-cell clones from COVID-19 patients [28]. They further demonstrated that the most potent amongst these antibodies was targeted against the RBD of the S protein, which was able to block the interaction of the S protein with ACE2 successfully. Yang et al. tested several binding inhibitor peptides, targeting the virus early attachment stages [29]. Others have observed a strong correlation between levels of RBD-binding antibodies and SARS-CoV-2 neutralizing antibodies in patients [22,30,31,32,33,34]. Thus, study of spike protein interaction with the ACE2 receptor can be of importance, for not only virus entry into cells, but also as a means of inhibiting virus infection of susceptible cells, development of vaccines, and detection of virus infection.

In this work, an optical-based time detection method incorporating the smartphone light source and a portable mini spectrometer for SARS-CoV-2 detection was developed, based on the ability to measure antigen-antibody binding interactions.

## 2. Materials and Methods

### 2.1. Optical Mini-Spectrometers

C11708MA from Hamamatsu/Japan [35] was used to convert the variable attenuation of light waves as they passed from end-to-end or reflected off substances into signals with spectral responses, ranging from 640 to 1010 nm. The wavelength reproducibility ranged between −0.5 and 0.5 nm and had a maximum of 20 nm FWHM spectra, under constant light conditions. The measurements were conducted with the room lights on. The distances among the light source, the spectrometer, and the sample holder were adjusted to eliminate any possible interference and to stabilize the spectrometer performance. Furthermore, the spectrometer was aligned with the light source and the sample cuvette to achieve a straight path of light.

### 2.2. Smart Mobile Phone

The smartphone light source was used as the main light source [36]. The mobile light emits lights with the spectral range from 380 to 740 nm. The maximum optical power was emitted at a wavelength of 623 nm. In this work, iPhone 8 was employed, though any smart phone can be used.

### 2.3. Nucleocapsid Protein

The SARS-CoV-2 nucleocapsid protein (Sino Biologicals, Cat no. 40588-V08B) [37] and its corresponding nucleocapsid antibody (Sino Biologicals Cat no. 40588-T62) [37] were used for the binding affinity experiments. The lyophilized protein was resuspended at a stock concentration of 0.25 mg/mL, according to the manufacturer’s instructions, in sterile water. The nucleocapsid rabbit polyclonal antibody was supplied at a stock concentration of 1 mg/mL.

### 2.4. The Receptor Binding Domain (RBD)

The receptor binding domain (RBD) of the SARS-CoV-2 spike protein (Sino Biologicals, Cat No 40592-V05H) [37] was expressed as a recombinant protein with the Fc region of mouse (mFc) at the C terminus end and its corresponding spike RBD antibody (Sino Biologicals, cat. no. 40592-T62) [37] was used for the binding affinity experiments. The RBD protein was prepared in sterile water at a stock concentration of 0.25 mg/mL, as per the manufacturer’s instruction. The spike RBD rabbit polyclonal antibody was prepared at a stock concentration of 1 mg/mL and diluted further for analysis.

## 3. Results and Discussions 

### 3.1. Experimental Design

**Figure 2:**
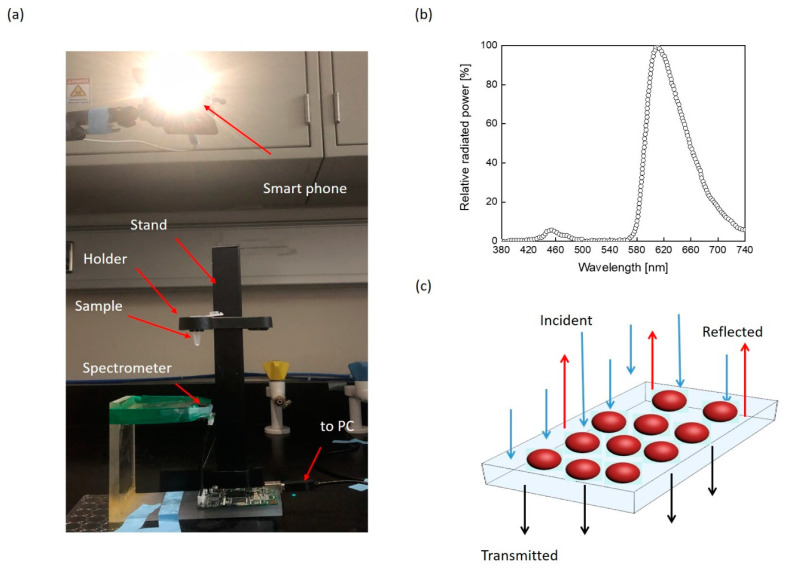
The proposed concept of optical detection and the experimental design: (**a**) the optical measurement setup is shown, consisting of a smart phone as a light source and the mini-spectrometer utilized to collect the light waves passing through the sample kept in the holder. (**b**) The smart phone power spectra vs. wavelength. (**c**) Illustration of the spectrometer detection principle.

The experimental setup utilized in this study is shown in Figure 2a, incorporating a mini spectrometer and a smart mobile phone that was employed as a light source with its power spectrum depicted in Figure 2b. The measured optical power of the beam exhibited maximum power at a wavelength of 623 nm [36]. The mini-spectrometer C11708MA (Hamamatsu/Japan) was used to measure the light intensity as it passed through test substances with spectral responses ranging from 640 to 1010 nm [35]. The wavelength reproducibility was between −0.5 and 0.5 nm and a maximum of 20 nm FWHM spectra, under constant light conditions. The sample under test was placed between the mobile light source and the mini-spectrometer, as shown in Figure 2a. The measurements were conducted with the room lights on. The distances among the light source, the spectrometer, and the sample holder were adjusted to eliminate any possible interference and to stabilize the spectrometer performance. Furthermore, the spectrometer was aligned with the light source and sample cuvette to achieve a straight path of light. Figure 2c illustrates the incident, reflected, and transmitted light intensities. The light intensities were linked through the Kirchhoff’s Law of Radiation [38], which correlates the optical absorbance, transmittance, and reflection, along with the incident wave.

**Figure 3:**
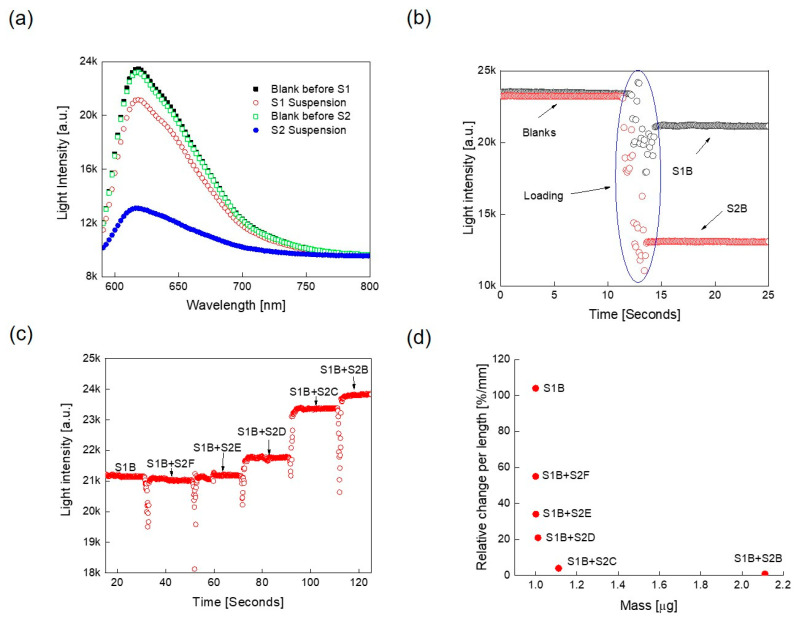
Optical measurements of the spike protein subunits S1 and S2: (**a**) measured responses for spikes proteins S1 and S2 at the highest concentration individually (S1B and S2B, respectively), along with their corresponding blanks. (**b**) Time domain measurements of the microcentrifuge tube, the blank (shown in gray circles) vs. water (red circles) at a wavelength of 623 nm. (**c**) Measured optical responses for the mixed protein samples vs. time. Samples S1B and S2B were at 5000 copies per ml, S2C, S2D, S2E, and S2F are the serial dilutions of S2B at 10-, 100-, 1000- and 10,000-fold, respectively. (**d**) Relative change in light intensity per light path vs. loaded mass. All optical responses were measured at 623 nm. Light intensity was measured as arbitrary units (a.u).

This experimental setup was first used to characterize the two spike proteins subunits, S1 and S2, which are encoded by all coronaviruses and, as mentioned, allow virus entry into susceptible cells (Figure 1b). Figure 3a shows the optical responses for both proteins along with their corresponding blank samples. The measured optical intensity changed from 600 to 750 nm, within the light source spectrum measured earlier in Figure 2b. The response of the blank samples was performed first, followed by the two protein suspensions, the responses to which were recorded individually as shown in Figure 3a.

Figure 3a reveals that S2 exhibited a higher “back scattering” and/or absorbance than S1. The response of the two blank samples was quite comparable, showing the reproducibility of the results. Since the maximum difference between the blank and the two protein samples was observed at 623 nm, this wavelength was chosen for further experimentation, which is also the wavelength at which the optical power of the smart phone is at its maximum.

### 3.2. Optimization of the Sample Reading Conditions

An initial test of this experimental setup revealed that it had one major drawback; i.e., when samples were loaded into the holder, the angle and position of the microcentrifuge tube changed, which affected the results obtained. To ensure that the results were reproducible, the measurements for the same samples were conducted over different days, and on each day, the setup was standardized, since the position of the mobile phone, spectrometer, and samples could vary. To overcome this caveat and have more consistence measurements without constant standardization, advantage was made of the ability of the spectrometer to provide light intensity measurements over time. Hence, after placing the microcentrifuge tube into the holder, the measurement mode started and the corresponding “blank” recorded. Then the sample was added after ~100 ms, while keeping the measurement mode on. Figure 3b illustrates the corresponding measurement profile for S1B and S2B individual samples suspended in water over time. Initially, a fluctuation in the light intensity was observed with time, as each sample was added to the tube, but then it stabilized with time. As expected, the blank exhibited the maximum measured light intensity, while the suspended samples showed lower light intensity than the blank once stabilized.

### 3.3. Test of the Spike Proteins Using the Proposed Experimental Set-Up

To test the proof-of-principle, initially a mixing experiment was conducted at a light wavelength of 623 nm. Towards this end, 250 μL of S1B protein solution was tested at the same maximum concentration at 5000 copies/mL followed by addition of the same amount of S2B. Figure 3c shows the light intensity (as arbitrary units, a.u.) with time as the protein samples were added to the *transparent measurement container* in a sequential manner. This was followed by the addition of 250 μL of ten-fold serial dilutions of the S2 protein at equal time intervals to the S1B + S2B samples. As can be seen from Figure 3c, with the addition of the S2 protein, the light intensity increased. The biggest increase was observed with the concentrated S2B sample followed by its ten-fold dilution samples S2C, S2D, S2E, etc., until S2F addition as a 1:10,000 dilution had no extra effect on the increase in light intensity, revealing the limit of detection of the assay (5000 molecules per mL × 250 µL × 1/10,000 = 125 molecule per mL). These results reveal that the ratio between the S1 and S2 protein concentration plays an important role in the light intensity levels measured. The ratio of S1 and S2 in the virus is the same since both originate from the cleavage of the S protein. However, the S1 subunit is expressed on the cell surface, while the S2 subunit is embedded in the lipid bilayer of the cell membrane; therefore, S2 is less available at the cell surface, which should affect light intensity less than S1, despite equal ratios. Table 1 lists the extracted parameters at specific time points. The relative change in light intensity per light path length is a constructed parameter that should correlate with the loaded mass (concentration) of the protein in a suspension.

Figure 3d shows the change in relative light intensity divided by the light path length vs. the total mass of the tested samples. As shown in Table 1, it reveals that, as the mass of the protein increased in our experimental system, the intensity of light also increased, revealing that length of the light path was directly proportional to the amount of protein in the sample.

**Figure 4:**
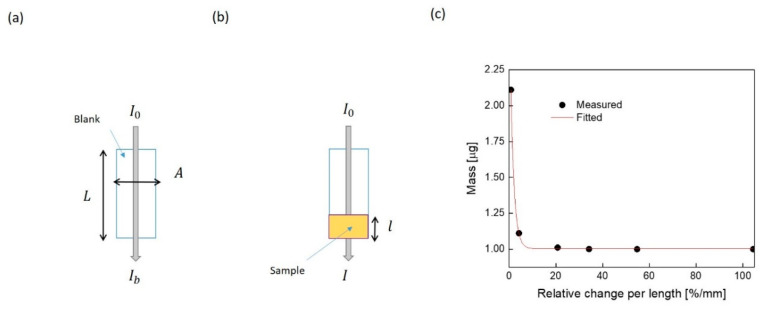
Illustration of light intensity and its path length: (**a**) the blank representation, and (**b**) light path length of the sample. L and A are the length and cross-sectional area of container, l is the light path length. $I_{0}$, $I_{b}$, and I are the incident, blank, and instantaneous sample intensities, respectively. (**c**) Loaded mass vs. relative change in light intensity per light-path length. The measured points were fitted with exponential function expressed by Equation (1) with the following parameters: *m_i_* = 1.003μ ± 2.68n, *m_f_* = 2.163μ ± 34.7n, and α-factor is 1.28435 ± 0.030. The other fitting model accuracy parameters are reduced Chi-Sqr, R-Square (COD), Adj. R-Square are 28.8 atto, 1 and 1, respectively, which indicates the best possible fit.

Figure 4a,b illustrates the definition of the light intensities and light path length. The smart mobile integrated light source emits a light intensity ($I_{0}$) that is the maximum intensity that can be measured in this experimental setup. The blank intensity ($I_{b}$) is the measured intensity that goes through the empty container responsible for holding the sample, such as the microcentrifuge tube. The instantaneous measured intensity (I) is the recorded light when it passes through the sample. This amount of light intensity strongly depends on the buffer in which the sample is solubilized/dissolved in, its composition, the light path length, the kind of the suspended analytes, and its size in the buffer. The light path length depends on the loaded amount of suspension inside the container. The path length varies from zero up to the container length (L). For a sample with a specific volume (V), the corresponding path length is equal to the volume over the cross-sectional area of the container (A). Equation (1) expresses the relationship between the relative change in light intensity per light path length and loaded mass (m), as follows:

(1) $$m=m_{i}+m_{f}e^{-\alpha \left(\Delta I/l\right)}$$

where $m_{i}$, $m_{f}$, and l are the initial mass of the buffer, the mass of the final suspension composite, and the light path length, respectively. α is the decay factor, unique for each control buffer. Its unit is in mm and could be correlated with the material absorptivity. ΔI is the relative change in light intensity expressed as follows:

(2) $$\Delta I=\left(1-I/I_{b}\right)\times 100\%$$

where I and $I_{b}$ are the instantaneous measured light intensity of the suspension and the corresponding blank, respectively. Figure 4c shows the relationship between mass and the relative change per length after fitting the measured points with the exponential function. As can be seen, with more sample volume, the path length increases and light intensity decreases; hence, the relative change decreases dramatically.

### 3.4. Test of Binding Interactions between Spike and ACE2 Using the Optical Assay

**Figure 5:**
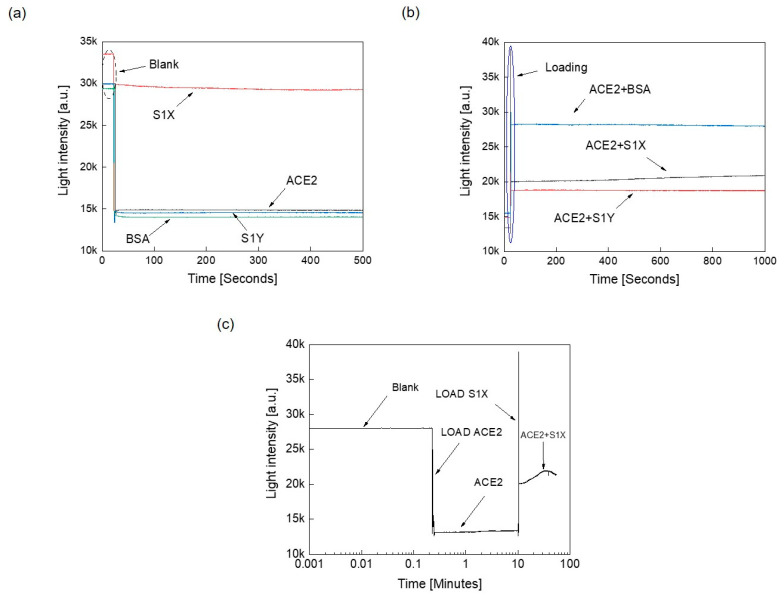
Optical detection of binding interactions between ACE2 and other proteins. (**a**) Measured light intensities over time for individual assessment of ACE2, S1X, S1Y, and BSA. (**b**) The measured mixed light intensities vs. time for ACE2 mixed with S1X, S1Y, or BSA. (**c**) The measured ACE2–S1X interaction profile for an extended time period.

After successful demonstration that our set-up could detect spike proteins in solution using light, we asked if light intensity could be used to characterize the binding interactions of the spike protein with the viral receptor ACE2. Towards this end, two different variants of the S1 subunit of the spike protein, S1X and S1Y, were tested (one form that could bind ACE2 with a much stronger affinity than the other one), along with a non-specific control protein—bovine serum albumin (BSA)—that should not bind to ACE2. These proteins were selected to demonstrate the detection of the binding process with ACE2 over time. The measurement process started with the blank, and after 200 s, 250 μL of ACE2 protein suspension was tested (Figure 5a). This process was repeated for S1X, S1Y, and BSA, and their responses to light were measured individually in the same manner as ACE2. The corresponding individual profiles of ACE2, S1X, S1Y, and BSA are depicted in Figure 5a, which showed a straight constant line over time. Next, each protein was mixed with the ACE2 separately to detect any possible binding effect. The measurements started with first loading the ACE2 in the blank container, then after 200 ms, the test protein was added to the ACE2 in solution. The responses of the various protein mixtures were read over a period of 15 min and are shown in Figure 5b.

Figure 5b shows the corresponding slopes that represent the change of the light intensities over time. The ACE2 + BSA and ACE2 + S1Y responses exhibited almost constant lines, suggesting highly reduced or lack of any interaction as observed when the proteins were tested individually Figure 5a. However, the ACE2 + S1X profile showed a linear straight line with the maximum-recorded slope. The corresponding light intensity line increased over time, suggesting an interaction between the SIX protein and the ACE2 receptor. We interpret this to mean that there was no protein–protein interaction if the slope of the line was zero; otherwise, protein–protein interaction occurred. Based on these observations, our results suggest that the S1X protein exhibits stronger interactions with ACE2, while BSA and S1Y had weaker interactions with ACE2. These observations are confirmed by the fact that S1X has a higher affinity for ACE2 (2 µg/mL S1X can bind 1.5–15 ng/mL ACE2), while S1Y reportedly has a much lower affinity (2 µg/mL S1B binds 0.5–8.7 ng/mL ACE2), as tested in enzyme-linked immunosorbent assays (ELISA) by the company that synthesized these proteins [39].

To explore the interaction and binding characteristics between ACE2 and S1X in more detail, the measurement time between the two proteins was extended over one hour, the results of which are plotted in Figure 5c. As can be seen, a nice “hump” was observed as an increase in arbitrary units (a.u.) with time that was not observed in the other protein mixtures tested, which we feel is indicative of the binding reaction between the two proteins.

### 3.5. Validation of the Optical Assay Using Known Antigen/Antibody Pairs

Next, we wanted to confirm our observations by using our optical system to detect protein–protein interactions using proteins that are well known to interact with each other. This was addressed by testing the molecular interactions between an antigen and an antibody, which is similar to the interaction between the spike protein and its receptor. Towards this end, two proteins were tested along with their specific antibodies: the first protein was the receptor-binding domain (RBD) of SARS-CoV-2 spike protein and its antibody and the other was the nucleocapsid protein (NCP) of SARS-CoV-2 and its antibody. Similar to the procedure described earlier, the two proteins were tested individually in our optical assay followed by addition of their corresponding antibodies that were mixed and then tested for their interactions.

**Figure 6:**
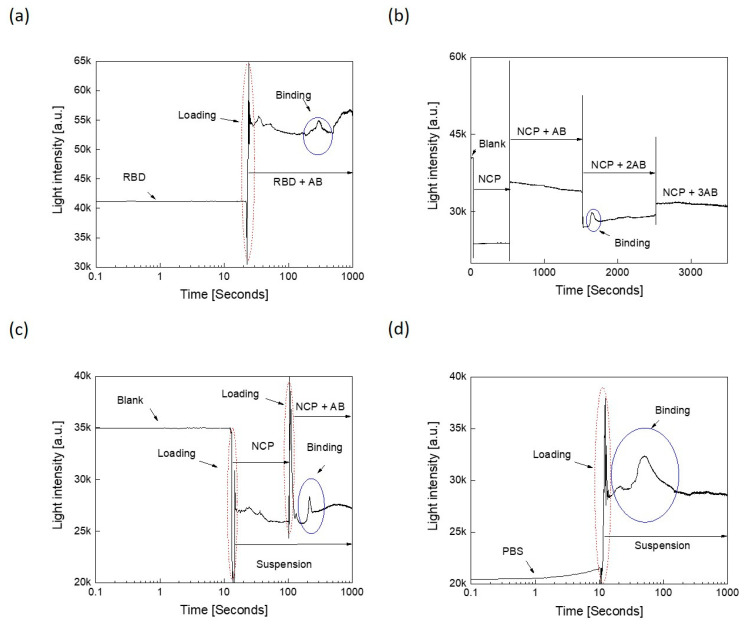
Optical detection of the binding affinities between: (**a**) the receptor-binding domain (RBD) of the spike protein with its antibody (AB), and (**b**) the nucleocapsid protein (NCP) and its antibody. The antibody was added again to NCP since no binding interaction was observed the first time. To confirm the result, the antibody was added a third time, but this time once again, the binding interaction was not apparent. (**c**) NCP binding with the antibody after mixing inside, and (**d**) NCP binding with the antibody after mixing outside. The interaction peak is circled.

Figure 6a shows the binding between RBD and its antibody. Upon the addition of the antibody, as observed earlier, an “interaction peak” was recorded (circled in the blue color). Similarly, Figure 6b shows the binding between NCP and its antibody. However, in this case, we realized that the binding effect occurred at specific antibody concentrations; thus, when the antibody was added first, no interaction peak was observed. Therefore, we added more concentrated antibody and upon its addition, the interaction peak was observed. The addition of more antibody did not allow detection of further interaction peaks, revealing that the protein–protein interaction took place at a specific concentration, and once the interaction had taken place, no further interaction took place. For a virus-based suspension, it is therefore suggested to use a fixed antibody concentration and serially dilute the virus suspension to conduct the binding measurements. Certainly, at a specific virus concentration, binding effect will appear in the form of an optical response.

Figure 6c,d illustrates the corresponding optical responses for the NC protein and its corresponding antibody, when they were mixed inside (Figure 6c) or outside (Figure 6d) the microcentrifuge tube, respectively. Inside mixing means that the protein was added to the tube and the antibody was added after 10 s, while in the outside mixing scenario, both the protein and antibody were mixed prior to being loaded in the tube for optical measurements. As can be seen, the binding response could be detected in each case in the form of appearance of the hump. However, this “hump” was a lot more pronounced when the protein and the antibody were mixed prior to testing than when they were added sequentially. This is good news for the real-life scenario, where in a patient sample, the antibody should be already bound to the viral or bacterial antigen at the time of detection.

### 3.6. Test of the Optical Detection Assay Using a Solid Support

**Figure 7:**
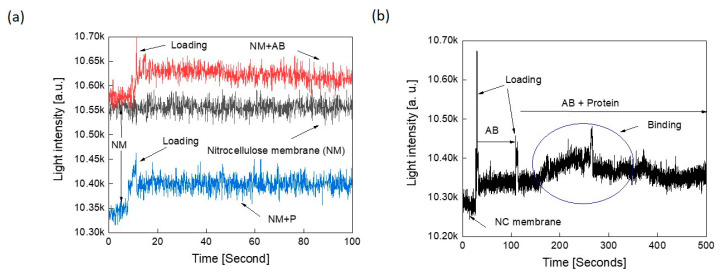
Test of protein–protein interaction measurements on solid support: (**a**) optical responses on nitrocellulose membrane (NM) alone, nitrocellulose membrane and spike protein (NM + P), and nitrocellulose membrane and antibody to spike protein (NM + AB) alone. (**b**) Optical responses to spike protein–antibody binding on the nitrocellulose membrane.

The nitrocellulose membrane is a popular matrix that is frequently used due to its high protein-binding affinity with a pore size of 0.25–0.45 µm in paper-based diagnostics. Protein molecules usually bind to the nitrocellulose membranes through hydrophobic interactions [40]. Due to the ease of their handling, cheap cost, and the presence of hydrophobic interactions between them and the suspended proteins, we tested whether the binding between the SARS-CoV-2 spike protein and antibody could be detected optically when both were added to each other on the nitrocellulose membrane. Using the experimental setup detailed in Figure 2a, the optical responses for nitrocellulose membrane, nitrocellulose membrane and spike protein alone, nitrocellulose membrane and antibody against spike protein alone, and nitrocellulose membrane spike protein–antibody were measured. Figure 7a shows that both the antibody alone and spike protein alone exhibited higher light intensity than the nitrocellulose membrane alone with almost a straight line with a constant slope over a time period of 10 s. The on-paper measured optical responses exhibited fluctuations as in the samples measured using microcentrifuge tubes. This implies that these fluctuations are not due to any interactions; rather, they are due to the spectrometer conversion process [41].

Figure 7b summaries the interaction measurements, which start with the membrane alone. After 20 s, the antibody suspension was loaded on the membrane and measurements were conducted up to 100 s. Next, the spike protein sample was loaded and measurements were continued up to 500 s. As revealed from Figure 7b, the interaction peak clearly appeared as circled in blue. It is worth noting that the membrane size, shape, and charge of biomolecules, pH, and viscosity of the control buffer, as well as the composition influences the corresponding optical response and binding interactions and must be carefully standardized [42].

### 3.7. Role of Electric Current in Disrupting Protein-Protein Interactions

**Figure 8:**
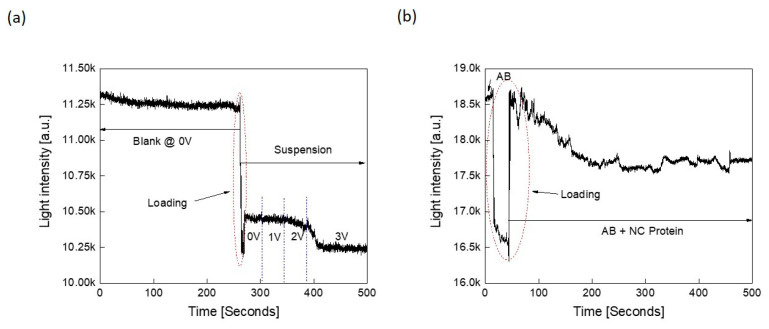
Opto-electrical measurements: (**a**) Measured NC protein optical response versus time at different DC bias voltages. (**b**) Binding measurements between NC protein and its corresponding antibody after subjected the solution to an electric field.

Finally, we studied the effect of direct current (DC biasing) on the ability of two proteins to bind specifically. This was achieved by subjecting the NC protein solution to DC voltage bias, as depicted in Figure 8a. An applied bias should result in an induction of current across the suspension. If this current is high enough, it should have the potential to destroy the protein physiology and functionality, resulting in the loss of specific protein-protein interactions. To test this hypothesis, the NC protein solution was loaded in an electroporation cuvette (rather than a microcentrifuge tube) that incorporates two electrodes with a volume of 0.5 mL and a separation distance of 0.4 cm. This should result in a breakdown electric field of 7.5 V/cm. At this field onwards, the binding between the protein and the antibody should be affected. Above this field, the sample should be incapacitated for binding. Figure 8a reveals that the optical response decays slowly with the application of DC bias. At 3 volts DC bias, the optical response decays with a considerable step, and increasing the DC bias further should burn the suspension and destroy it.

The breakdown field depends on the electrical characteristics of both the buffer and the analyte such as proteins, viruses, etc. To explore this further, the suspension of protein was subjected to 3 V for 1 min and then the antibody to NC was added to the NC protein solution. The corresponding measured response is shown in Figure 8b. The measured response was observed to be noisy and did not show a clear binding effect when compared with Figure 6c that reported the optical response for the same protein and antibody without the application of DC bias. It is worth mentioning that it may be possible to create a corresponding vaccine for a disease by subjecting the target viral protein to DC bias which will affect its function and destroy its physiology (denature it) and communicability (binding interactions). Furthermore, the proposed optical detection in time domain can also be used for monitoring and detecting the efficiency of vaccine process development.

## 4. Discussion

This study establishes the proof-of-principle that optical methods can be used to detect specific SARS-CoV-2 spike proteins or their subunits (S, S1, and S2) as well as their interactions with the ACE2 receptor in solution, whenever present (Figure 3 and Figure 5). The principle was further validated by testing specific protein–protein interactions by testing two viral protein–antibody pairs (RBD and NC proteins with their specific antibodies) and testing them either in solution (Figure 6) or on a solid matrix (Figure 7). Finally, it was shown that application of a weak current into the system could lead to the disruption of NC protein–antibody interactions, which could be optically detected (Figure 8). In other words, our technique could be used not only to detect specific SARS-CoV-2 spike protein–receptor interactions, but also result in destruction of protein interactions important for virus replication; thus, inhibiting rate of infection. The proposed detection method can be performed within minutes, without the need to biochemically label the proteins. This system can be used to develop novel optical-based detection tests for any virus in a specific and sensitive manner, as long as one specific protein partner is available in the solution or on a solid support that can interact specifically with a specific viral protein. For instance, in the case of SARS-CoV-2, one could use either an antibody to the spike protein or the ACE2 protein to determine whether a particular patient sample may have the virus.

Figure 1c shows the spikes distribution suspended in a sample. As illustrated, the spike proteins are randomly distributed and exhibit Brownian motion by default [43]. The same scenario applies to the distribution of ACE2 as illustrated in Figure 1d [44]. If specific binding occurs between the spike proteins and ACE2, as represented by Figure 1e, their binding distribution should still exhibit random Brownian motion. Nevertheless, the SARS-CoV-2 spike protein binds with human ACE2 protein with a specific binding energy that has been measured and is estimated to be nearly −58.55 ± 8.75 kmol^−1^ [45,46]. A conformational change occurs in the ACE2 receptor protein after binding with spike protein fragment [46]. Ov et al. showed that the refraction index changes due to the binding interactions after the virus-antibody incubation process [47]. To detect binding of living cells and viruses with potential drugs, they proposed a novel label-free real time approach, incorporating long-range surface waves on a one-dimensional photonic crystal surface along with microfluidic channel technology [48].

The photoelectric effect theory combines kinetic energy, binding energy, and photon energy all three of which are correlated through the theoretical physics fundamentals and principles [44]. Hence, if the generated photon energy due to the binding interactions is sufficient, it could give rise to light intensity at a specific wavelength. Wang et al. discussed the enhancement of receptor binding of SARS-CoV-2 through networks of hydrogen-bonding and hydrophobic interactions [46]. They provided explanations to better understand the structural and energetic details responsible for protein–protein interactions between the host receptor ACE2 and SARS-CoV-2. Their simulations reveal that both electrostatic complementarity and hydrophobic interactions are critical to enhance receptor binding and escape antibody recognition by the RBD of SARS-CoV-2. Ortega et al. conducted an in silico analysis to study the role of changes in SARS-CoV-2 spike protein during interaction with the ACE2 receptor. They concluded that the binding energy generated during the SARS-CoV-2 spike and ACE2 interactions can be reduced due to mutations in the sequence of the spike protein [18]. Dahal et al. demonstrated that binding probability increases with antibody concentration and the stability of protein [48].

Based on these observations, we believe that a “hump or spike” in light intensity is observed when a specific molecular interaction takes place between two proteins. This is mainly due to the physiochemical properties of the proteins that relate to binding affinity in the contact surface area, which incorporates the association/dissociation process [39].

**Figure 9:**
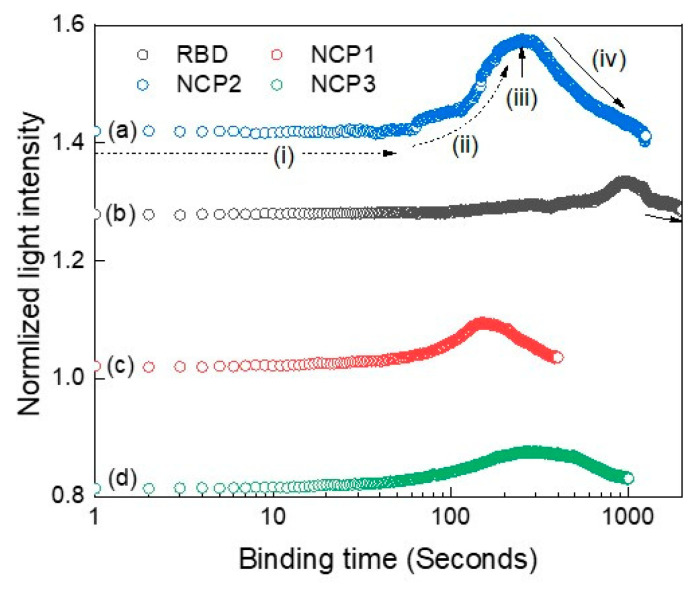
Optical profiles for normalized interactions vs. binding time under different conditions for RBD and NCP. (**a**) NCP with its antibody at the maximum concentration of 22 μg per mL; (**b**) RBD with its antibody; (**c**) NCP with its antibody at a 1:10 dilution; (**d**) NCP with its antibody at 1:10 with outside mixing.

As revealed from the corresponding binding measured light intensity profiles, they exhibit Gaussian-like peaks. Wang et al. demonstrated in their study that the molecular binding at the single molecule level displays such a peak [40]. Interestingly, Kozono et al. monitored the real-time Brownian motion and fitted it with Gaussian function [41]. The fitting parameters of the distributions can provide many features of the binding interactions [42]. This could provide a quantitative signature or characterization of a specific antigen binding to a specific antibody, such as intrinsic specificity and binding rate. It is also proven that the probability of the binding free energy to be Gaussian distributed near the mean and exponential-like distributed in the tail [43]. Figure 9 shows the binding interaction over the corresponding time intervals for RBD and NCP proteins with their corresponding antibodies under different conditions, as described in Figure 6. Similar interaction peaks were observed in each case, except they varied in the time of appearance and extent of light intensity. The time slot denoted by (i) represents the time just before the interaction occurs. As the interaction starts, the corresponding optical profile ascends incrementally as indicated by (ii) due to the increase in binding events, releasing more photons energy. The peak pointed by (iii) occurs at the maximum event of binding between antigens and their antibodies. The height of the peaks indicates stronger interactions and vice versa. The profile then descends until the end as the binding events become less, and no further interactions occur at the end, as illustrated by (iv). The distance in time to maximum peak reflects the speed of the binding interactions; thus, the earlier the peak appears, the faster the binding interaction takes place. As can be seen in Figure 9, the speed of NC protein binding to its antibody took place between 100 and 1000 s, irrespective of the dilution of the proteins.

Next, we analyzed the interaction profiles of these samples further by fitting them to Gaussian function. Table 2 lists their corresponding fitting parameters. The most important parameters are the width and the maximum peak amplitude. Base and center parameters represent the offset level and the maximum peak location, respectively. These two parameters provide minor information and could be set to fixed values, such as zero in all profiles. The multiplication of the maximum amplitude with its corresponding width can be utilized as an indicator to describe the speed of interaction. This “indicator” is listed in Table 2, last row. Accordingly, NCP2 exhibited the fastest binding, while NCP3 exhibited the slowest binding with the arrangement from fast to slow being as follows: NCP2, NCP1, RBD, and NCP3.

### Practical Applications of the Proposed Technique

The optical approach presented in this study can be easily turned into a functional working system to detect SARS-CoV-2 or any other viral or bacterial pathogen against which an antibody is available that can detect it sensitively. Currently, there are several available handheld portable spectrophotometers, which are compact and lightweight, and equipped with wireless communication system for data transmission. The data can be received by the smart phone through Bluetooth or Wi-Fi technologies equipped with a mobile app designed to process the received optical profiles over time. The collected optical profiles vs. time can then be processed immediately using the smart phone processor and computational resources. The results can be displayed on the same smart phone immediately as well. The antibody could be coated on flexible strips and kept inside packs with medium. These strips can be used directly for loading the nasal swab in-position within a fabricated holder using 3D printing technology. The 3D printed holder can be designed to integrate the portable sensor and the smart phone as well. The concept of strips can be further used to detect different specimens taken from blood, breath, urine, nasal swabs, stool, etc. For example, the subject can breathe, exhale, or sniff into a device with a probe coated with antibodies. If binding occurs, the integrated device should be able to pick up the interaction, confirming that the patient is infected with the virus to which the antibodies are directed against.

The current methodology provides a rapid, reproducible, and accurate detection mechanism that can be used to create home-based COVID-19 detection tests that can be used by anyone. The authors envision that such tests would not require any laboratory setting, and could be performed using test strips coated with antibodies without prior sample processing. Interestingly, our approach does not require any electrode patterning, which makes it the best fit for massive production and high-volume use. It can be adapted as a point-of-care testing platform with high-throughput to be used in schools, airports, malls and public services places as well. The cost per test is expected to be less than one dollar, which will make it competitive with current market price. The detection platform can be easily equipped with standard electronic data transmission systems to transmit and process data in place and share it with family, doctors, and hospitals over wireless transmission. Furthermore, the platform can be deployed in high-risk areas with ease-of-use and clear steps to load the specimen and easy to understand instructions to operate.

Furthermore, when compared with other detection concepts and methodologies, the presented approach can distinguish between influenza and coronaviruses. ACE2 binds directly to the viral spike protein, while ACE2 plays an important role in acute lung injury induced by influenza viruses [49], which can be correlated with disease severity [50]. Hence, the proposed methodology can be used in reverse where the spike protein can be used for ACE2 detection.

## 5. Conclusions

In summary, this study provides proof-of-principle for an optical-based, quick, simple, and sensitive screening technology for the detection of SARS-CoV-2. It is based on the principle that when light passes through a sample, interactions between the photons and sample occurs within a specific range of *wavelengths.* The current approach utilizes a smartphone light source and a portable mini-spectrophotometer to convert the variations in light intensity into measured signal. The optical responses could further be analyzed using the principle component analysis technique to enhance and allow precise detection of the specific target in a multi-protein mixture. This approach can be further developed to accommodate mass screening that should provide fast and accurate positive or negative test results.

## Figures and Tables

**Table 1:** List of measured and extracted parameters.

Sample Description	Light Intensity (a.u.)	Length of the Light Path (mm)	Mass of Protein Tested (μg)	ΔI_r_ Per Length (%/mm)
S1B	21,215	0.11111	1	104
S1B + S2F	21,080	0.22222	1.0001	55
S1B + S2E	21,265	0.33333	1.0011	34
S1B + S2D	21,785	0.44444	1.0111	21
S1B + S2C	23,440	0.55556	1.1111	4
S1B + S2B	23,875	0.66667	2.1111	0.8

**Table 2:** Fitting parameters for interaction profiles depicted in Figure 9.

Fitting Parameters	NCP3	RBD	NCP2	NCP1
Base	1.43	1.29	1.03	0.83
Center	302	996	181	380
Width	130	252	68	194
Amplitude	0.13	0.04	0.06	0.05
Indicator	16.9	10.08	4.08	9.7

## Data Availability

Not Applicable.
